# Oxytocin modulation of the insular cortex: implications for social cognition and neurodevelopmental disorders

**DOI:** 10.3389/fncir.2026.1791625

**Published:** 2026-03-27

**Authors:** Shuhei Fujima, Masaaki Sato

**Affiliations:** 1 Department of Physiology and Cell Biology, Kobe University School of Medicine, Kobe, Japan; 2 Faculty of Applied Biology, Kyoto Institute of Technology, Kyoto, Japan

**Keywords:** autism spectrum disorder, contextual modulation, emotional mirroring, insular cortex, interoception, oxytocin, social cognition

## Abstract

Social cognition relies on the integration of sensory information, emotional cues, and internal bodily signals to guide behavior toward others. The insular cortex (IC) is anatomically and functionally well positioned to support this integration, as it receives interoceptive input and connects sensory, limbic, and autonomic systems. Accumulating evidence across species suggests that the IC contributes to social behavior through at least two complementary modes of processing: emotional mirroring, which links observed social cues to internal affective states, and contextual modulation, which adjusts social behavior according to familiarity, prior experience, and internal state. In this Mini Review, we discuss how neuromodulatory systems shape these modes of IC processing, with a particular focus on oxytocin (OXT). In rodents, OXT signaling within the IC influences social affective behaviors under specific social conditions, whereas human studies report heterogeneous and context-dependent effects of OXT on IC activity. Altered IC function and OXT signaling have also been implicated in neurodevelopmental disorders characterized by social deficits, including autism spectrum disorder. We propose that OXT modulates IC function in a context- and state-dependent manner, shaping social cognition by influencing how interoceptive, emotional, and contextual information is integrated.

## 1 Introduction

Social cognition requires the integration of sensory inputs, emotional signals, and internal bodily states to guide appropriate behavior toward others. During social interactions, individuals must infer others’ emotions, distress, and familiarity, and flexibly update their responses. Although many brain regions involved in social and emotional processing have been identified, how interoceptive signals contribute to social cognition at the neural level remains incompletely understood.

The IC is well positioned to address this problem, as it receives interoceptive input and is anatomically connected with sensory, limbic, and autonomic systems (Gogolla, 2017; Sato et al., 2023). Consistent with this position, the IC has been implicated in diverse functions, including taste (Yaxley et al., 1990), pain (Geuter et al., 2017), visceral sensation (Aziz et al., 2000), addiction (Contreras et al., 2007), and empathy-related processes (Wicker et al., 2003; Singer et al., 2004; Gu et al., 2012). However, these functions have often been discussed separately, and it remains unclear how they relate to one another within a common framework of social cognition, or how bodily signals processed in the IC contribute to the interpretation of others’ emotional states.

Recent studies further suggest that neuromodulators influence IC processing during social behavior. In particular, OXT has attracted attention because of its established roles in social interaction (Rogers-Carter et al., 2018) and its receptor expression in the IC (Sharma et al., 2019). Altered IC function (Sabatino et al., 2013; Odriozola et al., 2016) and OXT signaling (Saito et al., 2014; Zhang et al., 2016; Wang et al., 2021) have also been reported in neuropsychiatric conditions associated with social deficits, including autism spectrum disorder (ASD), although findings are heterogeneous. In this Mini Review, we therefore ask how the IC integrates interoceptive signals with social and emotional information, how this integration is influenced by OXT, and how disruptions of these processes may contribute to social impairments in neurodevelopmental disorders.

## 2 Main text

### 2.1 Emotional mirroring in the IC

**FIGURE 1:**
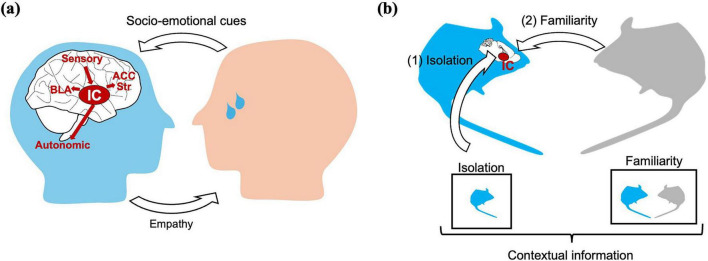
Insular cortex (IC) functions in socio-emotional and contextual aspects of social behavior. **(a)** Interoceptive simulation hypothesis. Social–emotional cues (e.g., pain, distress, and disgust) are first processed in sensory and affective regions and are then remapped onto the observer’s own interoceptive representations along the IC hierarchy. The posterior IC encodes primary bodily states, the mid-IC integrates observed and internal cues, and the anterior IC generates an “interoceptive simulation” that supports affective empathy. This simulated state is conveyed to downstream regions, including the anterior cingulate cortex (ACC), striatum (Str), basolateral amygdala (BLA), and autonomic centers, to guide empathic and social behavior. **(b)** Contextual and state-dependent social functions of the IC. Animal studies indicate that the IC also contributes to social behavior by integrating contextual information. (1) Social isolation enhances preference for novel conspecifics in an IC-dependent manner. (2) Social familiarity and bonding, in which the IC encodes familiarity and modulates social preference. Together, these functions highlight the integrative role of the IC in mapping contextual and internal state information onto social behavior.

The IC is involved in social behavior partly through emotional mirroring (Figure 1a), which refers to processes by which the brain responds to the emotional states of others (Singer et al., 2004; Gu et al., 2012). In many cases, this type of processing is thought to support empathy-related functions, such as responding to another individual’s pain or distress (Bernhardt and Singer, 2012; Gu et al., 2013). From this perspective, emotional mirroring involves linking external social cues to internal emotional or bodily responses in the observer (Figure 1a).

Human studies provide evidence that the IC participates in such socioemotional processing. Activity in the IC increases both when individuals experience pain themselves and when they observe others in pain (Singer et al., 2004), and lesions in this region reduce sensitivity to others’ pain (Gu et al., 2012). The IC is also activated during facial expression imitation (Carr et al., 2003) and during both the experience and observation of disgust (Wicker et al., 2003). These findings suggest that the IC is engaged when emotional states are shared or reflected during social interaction. Similar functions have been reported in non-human primates. In macaques, affiliative touch such as caressing evokes pleasant internal states (Grandi and Gerbella, 2016), and electrical stimulation of the IC can induce affiliative behaviors in rhesus monkeys (Caruana et al., 2011). These results indicate that the involvement of the IC in affective aspects of social behavior is not unique to humans.

Rodent studies further support a role for the IC in emotion-related social processing. The IC is required for emotion recognition (Rogers-Carter et al., 2018, 2019) and for detecting conspecifics undergoing inflammatory states (Rieger et al., 2022, 2023; Djerdjaj et al., 2023). At the cellular level, multiple neuronal populations contribute to IC activity during social interaction. Both excitatory neurons and inhibitory interneurons, including parvalbumin-positive and vasoactive intestinal peptide (VIP)-positive interneurons, respond to social cues in the mouse IC (Miura et al., 2020; Ramos-Prats et al., 2022; Fujima et al., 2025). However, the activation of these neurons during social behavior does not necessarily imply that they form a dedicated circuit for emotional mirroring. Rather, the same neuronal populations may participate in different types of processing depending on the situation. For example, IC ensembles can be recruited in a state- and context-dependent manner, and social representations within the IC are dynamically reorganized with experience (Glangetas et al., 2024; Sato et al., 2025; see Section “2.2 Contextual integration and state-dependent social behavior”).

Overall, these findings suggest that the IC contributes to emotional mirroring by responding to socially relevant cues and linking them to internal emotional or bodily responses. This process may particularly support empathic aspects of social behavior, although the precise neural computations involved remain to be fully clarified.

### 2.2 Contextual integration and state-dependent social behavior

In addition to emotional mirroring, the IC is also involved in social behavior through contextual integration. In this case, social actions are influenced by factors such as familiarity, prior social experience, and internal state (Figure 1b). This type of processing is particularly relevant for social behaviors that do not directly depend on sharing others’ emotions, including novel–familiar discrimination and familiarity-based judgments (Kim et al., 2023; Min et al., 2023).

Contextual factors such as familiarity or social history are not simply background variables that accompany social behavior. Instead, they can change physiological responses during social encounters, for example by affecting arousal level or autonomic tone. As a result, the bodily signals present during social interaction may differ depending on the context, which in turn may influence how social information is processed in the IC.

We previously reported that a subset of excitatory neurons in the IC, referred to as “social cells,” is activated during interactions with conspecifics (Miura et al., 2020). Repeated interactions with the same individual reduce the proportion of these responsive neurons (Sato et al., 2025), suggesting that the IC responds differently to novel and familiar social partners. Consistent with this, suppression of IC activity impairs the ability to discriminate between novel and familiar conspecifics (Min et al., 2023), indicating that the IC is required for familiarity-based social discrimination.

The contribution of the IC to contextual integration also depends on internal state. Under baseline conditions, suppression of IC activity does not strongly affect social interaction, as mice still prefer a novel conspecific over a novel object (Min et al., 2023). In contrast, after a period of social isolation, manipulation of IC neurons markedly reduces social approach behavior (Glangetas et al., 2024; Hiyoshi et al., 2025). These findings suggest that the IC becomes particularly important when social behavior needs to be adjusted according to internal conditions, such as increased social motivation following isolation.

Taken together, these results indicate that the IC should not be viewed only as a region involved in empathy. Instead, it appears to participate more generally in integrating sensory, emotional, and contextual information to support social behavior. Emotional mirroring and contextual integration may therefore represent two related modes of IC processing that operate depending on the social situation and internal state of the animal.

### 2.3 OXT modulation of IC function

To understand how social behavior is regulated by the IC, it is important to consider the role of neuromodulatory systems. Among these, OXT has attracted particular attention given its well-established involvement in social behavior and its ability to influence cortical processing. OXT receptors (OXTR) are expressed in the IC in rodents (Sharma et al., 2019), although their precise distribution across layers and cell types has not been fully characterized (Newmaster et al., 2020). Notably, direct OXT inputs from the hypothalamus to cortical regions, including the IC, appear relatively sparse and restricted, suggesting that these hypothalamic projections provide only a limited anatomical basis for OXT modulation of IC activity (Knobloch et al., 2012; Son et al., 2022; Kojima et al., 2024). Within the hypothalamus, OXT is produced primarily in the paraventricular nucleus (PVN) and supraoptic nucleus (SON); however, the specific hypothalamic sources of OXT input to the IC are not fully resolved. Recent studies reported that a subset of PVN OXT neurons projects to the IC (Gamal-Eltrabily et al., 2020; Li et al., 2024). In contrast, direct projections from the SON to the IC have not been firmly established, and thus the relative contributions of PVN versus SON to IC OXT signaling remain uncertain.

Beyond direct axonal projections, cortical OXT signaling has been proposed to arise through diffusion/volume transmission via the cerebrospinal fluid (CSF) and/or peripheral-to-central routes (Jones et al., 1983; Veening et al., 2010; Zheng et al., 2014; Yamamoto et al., 2019). At present, the relative contribution of direct axonal routes versus CSF- and/or peripheral-to-central routes to cortical OXT–OXTR signaling remains uncertain, and both routes should be considered when interpreting OXT effects in the IC. Moreover, OXT has been implicated in both aversive aspects of social behavior, such as social avoidance (Osakada et al., 2024), and rewarding aspects, including maternal (Carcea et al., 2021) and prosocial behaviors (Rogers-Carter et al., 2018). However, it remains unclear which OXT-sensitive neuronal populations preferentially support aversive versus rewarding social behaviors. Future studies will be required to clarify how OXT-dependent modulation of these distinct social behavioral components relates to specific hypothalamic sources and delivery routes.

Arginine vasopressin (AVP) is closely related to OXT and shows cross-reactivity with the OXT system. There are three AVP receptors (AVPRs): AVPR1a, AVPR1b, and AVPR2. OXT and AVP can cross-activate each other’s receptor systems, particularly via AVPR1a (Carter, 2017). AVP signaling has been implicated in the regulation of social behaviors, suggesting that AVPR expression in the IC may also contribute to neuromodulatory control of social processing, alongside OXT–OXTR signaling. A recent report described relatively high AVPR1a expression in the IC compared with other cortical regions in mice (Gumerova et al., 2025). In rats, AVPR1a expression in the IC has been reported to be lower relative to OXTR expression (Dumais and Veenema, 2016; Smith et al., 2017). These findings suggest that AVPR-mediated signaling may also influence IC function, although its contribution may be less prominent than that of OXTR-mediated signaling. Such species differences in receptor expression, together with OXT–AVPR cross-reactivity, highlight the need to interpret IC neuromodulation with caution when comparing studies across experimental conditions and species.

Functional studies in rodents support a role for OXT signaling within the IC during social and affective behaviors. Pharmacological blockade of OXTR in the IC impairs social affective functions, including emotion-recognition abilities in rats (Rogers-Carter et al., 2018). At the circuit level, OXTR expression has been reported in subsets of GABAergic neurons in the IC, suggesting that OXT may act, at least in part, by modulating local inhibitory microcircuits (Gamal-Eltrabily et al., 2020). More recently, social exclusion–related paradigms have been linked to OXT signaling in the IC, and blocking OXTR within the IC enhances pain responses toward socially excluded individuals, indicating a role for OXT in shaping how others’ emotional states are represented under socially challenging conditions (Tye et al., 2025).

Human studies provide additional, but less consistent, evidence for OXT modulation of IC activity. Intranasal OXT administration has been reported to reduce IC responses during socio-emotional tasks, including emotional perception and embarrassment-related processing (Geng et al., 2018; Ma et al., 2020). In contrast, other studies have reported increased activity in the left IC in response to socially salient stimuli, such as infant crying, following OXT administration (Riem et al., 2011). These mixed findings suggest that OXT does not exert a uniform excitatory or inhibitory effect on IC activity, but instead modulates IC engagement in a manner that depends on social context and task demands.

In humans, however, direct evidence for OXTR expression in the IC remains limited. Available postmortem and transcriptomic studies suggest that OXTR expression in cortical regions, including the IC, is relatively sparse and variable (Loup et al., 1991; Boccia et al., 2013), highlighting potential species differences and methodological constraints when interpreting OXT effects observed in human neuroimaging studies. Moreover, given OXT–AVPR cross-reactivity, differences in AVPR expression may also contribute to divergent human findings beyond OXTR expression alone. This uncertainty may partly account for the heterogeneous effects of OXT reported across human studies.

**FIGURE 2:**
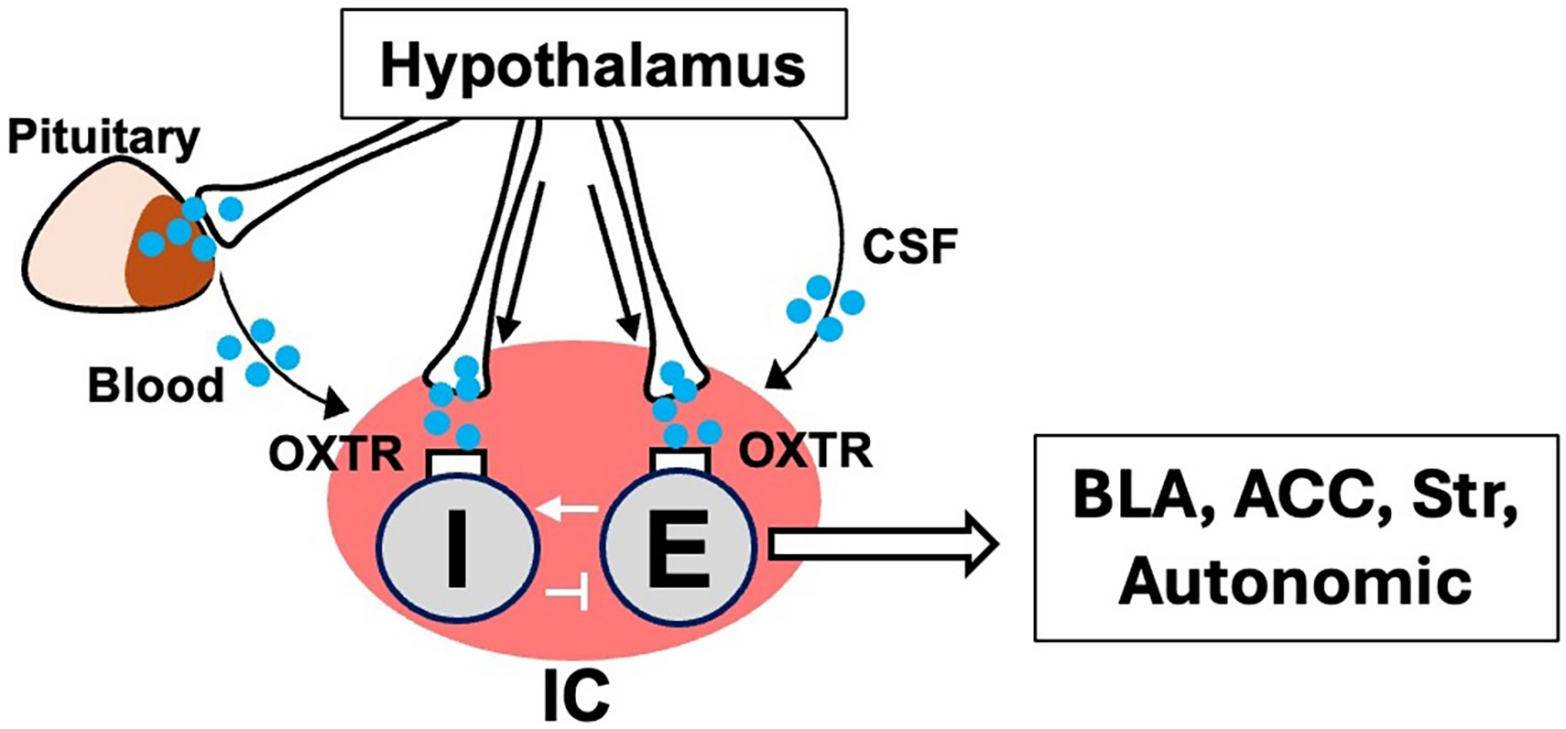
Proposed delivery routes and mechanisms of OXT modulation in the IC. OXT (blue dots) signaling in the IC is proposed to arise through multiple delivery routes. Sparse hypothalamic projections to the IC have been reported, predominantly from the PVN. Additional non-axonal routes have been suggested, including diffusion/volume transmission via the cerebrospinal fluid (CSF) and peripheral-to-central pathways via the blood. The relative contribution of axonal versus CSF- and blood-mediated routes remains uncertain. Within the IC, OXT can act on its receptors (OXTR) expressed in excitatory (E) and inhibitory (I) neurons, potentially modulating circuit activity and shaping the integration of interoceptive, emotional, and contextual information during social behavior.

A key unresolved question is how OXT alters IC computations at the circuit level (Figure 2). At the cellular level, OXTR activation engages intracellular signaling pathways, typically involving G_*q*/11_–PLC signaling and downstream Ca^2+^- and PKC-dependent modulation of membrane function (Gimpl and Fahrenholz, 2001). These pathways can modulate the excitability of OXTR-expressing neurons across multiple cell classes, including pyramidal neurons in hippocampal CA2 (Tirko et al., 2018) and principal neurons in the IC in a PKC-dependent manner (Rogers-Carter et al., 2018). At the circuit level, such cell-type-specific changes in excitability–potentially in both excitatory neurons and inhibitory interneurons–could be expressed as a modulation of the excitation–inhibition balance, thereby changing the signal-to-noise ratio of socially relevant interoceptive signals (Marlin et al., 2015; Oettl et al., 2016; Maldonado et al., 2021). Such modulation could influence the strength with which others’ emotional states are mirrored in the observer. Another possibility is that OXT biases the recruitment of specific IC neuronal populations that encode social context or internal state, such as familiarity or social need, thereby supporting flexible, state-dependent social behavior. These mechanisms are not mutually exclusive, as changes in inhibitory control could influence which neuronal ensembles are engaged under particular social conditions.

Importantly, these proposed mechanisms provide a potential link between the two modes of IC processing described above. OXT may influence emotional mirroring by altering the salience of affective and interoceptive signals, while also affecting contextual modulation by adjusting how internal state information is weighted during social decision-making. In this view, OXT acts not as a simple switch for IC activity, but as a modulator of how social and interoceptive information is utilized within IC circuits.

Taken together, current evidence suggests that OXT modulates IC function in a context- and state-dependent manner. Although many questions remain regarding receptor distribution and circuit specificity, understanding how OXT shapes IC computations may help explain species differences in OXT effects and provide a framework for interpreting alterations in IC-related social processing observed in neuropsychiatric conditions.

### 2.4 IC–OXT dysregulation in autism spectrum disorder

Altered IC function has been repeatedly implicated in ASD, particularly in relation to social and emotional processing. Neuroimaging studies in humans have reported atypical IC activity during social and affective tasks in individuals with ASD, although the direction of these changes is not consistent across studies. Some reports describe increased IC responses during emotional or social processing (Sabatino et al., 2013; Odriozola et al., 2016), whereas other findings suggest reduced or context-dependent IC engagement (Anderson et al., 2010; Aoki et al., 2014), indicating substantial heterogeneity within the ASD population.

Evidence from animal models further supports a link between IC dysfunction and autism-related behaviors. In mouse models of ASD, reduced expression of inhibitory markers such as GAD65 and parvalbumin has been reported in the IC, suggesting an imbalance between excitation and inhibition within local circuits (Gogolla et al., 2014). Consistent with a causal contribution of the IC, direct manipulation of IC activity can modify autism-like behaviors. For example, deep-brain stimulation of the IC has been shown to improve social deficits in valproic acid–treated rats (Xiao et al., 2022), indicating that altered IC activity is not merely a correlate but may contribute to behavioral phenotypes.

Oxytocin signaling has also been implicated in ASD, providing a potential link between neuromodulation and IC dysfunction. Several studies have reported lower peripheral OXT levels in individuals with ASD (Zhang et al., 2016), although the relationship between peripheral measures and central OXT signaling remains unclear. Genetic studies further suggest an association between OXTR variation and IC structure or function. Specific OXTR single-nucleotide polymorphisms, such as rs2254298A, as well as higher OXTR polygenic risk scores, have been associated with reduced gray matter volume in the IC (Saito et al., 2014; Wang et al., 2021). In addition, increased methylation of the OXTR gene has been linked to heightened IC activity during social tasks (Puglia et al., 2015), suggesting that altered OXT signaling may influence IC processing in ASD.

Several conceptual frameworks have been proposed to account for socio-emotional processing in the IC, and these frameworks have been discussed in relation to potential mechanisms underlying social deficits observed in ASD. One is the interoceptive simulation model (Braadbaart et al., 2014; Oliver et al., 2018), which posits that atypical processing of bodily signals impairs the ability to generate appropriate affective responses to others, thereby affecting empathy and social understanding. Another is the predictive coding model (Gu et al., 2013), which emphasizes deficits in generating or updating predictions about others’ emotional states, leading to unstable or inaccurate social inference. Although these frameworks are often discussed separately, they are not mutually exclusive. Reduced precision or reliability of interoceptive signals could propagate through predictive hierarchies, resulting in impaired prediction of others’ emotions and intentions. Within this context, the IC is well positioned as a site where these deficits may converge. By integrating interoceptive signals with social and contextual information, the IC may support both emotional mirroring and contextual modulation of social behavior. Disruption of OXT signaling within IC circuits could therefore affect how bodily signals are weighted and used during social interactions, leading to context-inappropriate or inflexible social responses. Finally, these considerations may help explain why OXT-based interventions in ASD have shown modest and variable effects. While OXT administration can improve certain aspects of social behavior in some individuals, therapeutic outcomes are inconsistent, possibly reflecting heterogeneity in IC function, OXTR expression, or baseline internal state. A more detailed understanding of IC-specific OXT modulation may therefore be necessary to identify subgroups of individuals with ASD who are most likely to benefit from such interventions, as well as the conditions under which OXT enhances social cognition.

## 3 Outlook and conclusion

In this Mini Review, we discussed the role of the IC in social behavior from the perspective of emotional mirroring, contextual modulation, and neuromodulatory control by OXT. Across species, accumulating evidence suggests that the IC is involved in representing socially relevant information by integrating sensory cues, interoceptive signals, and contextual variables. Rather than serving a single function, the IC appears to support multiple modes of social processing that are recruited depending on social context and internal state.

A central theme emerging from recent work is that emotional mirroring and contextual modulation should not be viewed as mutually exclusive processes. Instead, they likely reflect different computational regimes implemented within overlapping IC circuits. Emotional mirroring emphasizes the coupling of observed social cues to internal affective and bodily representations, whereas contextual modulation highlights how familiarity, prior experience, and internal state shape social behavior. The IC, positioned at the interface of bodily signals and socio-emotional inference, is well suited to flexibly switch or balance these modes.

Oxytocin provides one example of how neuromodulatory systems may influence IC function. Evidence from rodent studies indicates that OXT signaling within the IC can alter social and affective behaviors, while human studies suggest more heterogeneous effects that depend on task and context. These findings support the idea that OXT does not simply increase or decrease IC activity, but modulates how interoceptive and social information are weighted and used within IC circuits. Differences in receptor distribution, circuit organization, and baseline internal state may further contribute to species-specific and individual variability.

Disruption of IC function and OXT signaling has been implicated in neuropsychiatric conditions characterized by social deficits, such as ASD. However, the heterogeneity observed in both IC activity and OXT-related effects highlights the need for a more nuanced framework that goes beyond global measures of activation. Future studies combining cell-type–specific manipulations, circuit-level recordings, and careful control of social context and internal state will be critical for clarifying how IC computations are altered in disease.

Looking forward, an important challenge is to link descriptive findings across species to mechanistic models of IC function. Integrating interoceptive awareness and predictive coding frameworks may provide a useful conceptual basis for understanding how bodily signals and social inference interact within the IC. Such approaches may also help identify conditions under which neuromodulatory interventions, including OXT-based strategies, are most effective.

In summary, the IC should be considered a flexible hub for social cognition, whose contribution depends on the dynamic integration of interoceptive, emotional, and contextual information. Elucidating how neuromodulatory systems shape these processes will be essential for advancing our understanding of social behavior in health and disease.
